# The Spatial Association of Gene Expression Evolves from Synchrony to Asynchrony and Stochasticity with Age

**DOI:** 10.1371/journal.pone.0024076

**Published:** 2011-09-02

**Authors:** Qi Wang, Jianhua Huang, Xinmin Zhang, Bin Wu, Xiaoyu Liu, Ziyin Shen

**Affiliations:** 1 Huashan Hospital, Fudan University, Shanghai, China; 2 National Engineering Center for Biochip, Shanghai, China; 3 Shanghai Tenth People's Hospital of Tongji University, Shanghai, China; Michigan State University, United States of America

## Abstract

For multicellular organisms, different tissues coordinate to integrate physiological functions, although this systematically and gradually declines in the aging process. Therefore, an association exists between tissue coordination and aging, and investigating the evolution of tissue coordination with age is of interest. In the past decade, both common and heterogeneous aging processes among tissues were extensively investigated. The results on spatial association of gene changes that determine lifespan appear complex and paradoxical. To reconcile observed commonality and heterogeneity of gene changes among tissues and to address evolution feature of tissue coordination with age, we introduced a new analytical strategy to systematically analyze genome-wide spatio-temporal gene expression profiles. We first applied the approach to natural aging process in three species (Rat, Mouse and *Drosophila*) and then to anti-aging process in Mouse. The results demonstrated that temporal gene expression alteration in different tissues experiences a progressive association evolution from spatial synchrony to asynchrony and stochasticity with age. This implies that tissue coordination gradually declines with age. Male mice showed earlier spatial asynchrony in gene expression than females, suggesting that male animals are more prone to aging than females. The confirmed anti-aging interventions (resveratrol and caloric restriction) enhanced tissue coordination, indicating their underlying anti-aging mechanism on multiple tissue levels. Further, functional analysis suggested asynchronous DNA/protein damage accumulation as well as asynchronous repair, modification and degradation of DNA/protein in tissues possibly contributes to asynchronous and stochastic changes of tissue microenvironment. This increased risk for a variety of age-related diseases such as neurodegeneration and cancer that eventually accelerate organismal aging and death. Our study suggests a novel molecular event occurring in aging process of multicellular species that may represent an intrinsic molecular mechanism of aging.

## Introduction

Aging is one of the most fundamental biological processes. It is distinctly characterized by the systematic and progressive decline of physiological functions in virtually all tissues or organs with increasing age. This eventually contributes to an increased risk for pernicious diseases such as neurodegeneration and cancer [1]. Thus, the aging process of complex multicellular organisms is associated with the dynamic evolution of the underlying sophisticated coordination mechanisms among tissues or organs. During the past decades, researchers have explored molecular properties of the aging process in multiple tissues to gain global insight into its coordination, and to investigate specific and common molecular mechanisms that occur in different tissues [2]–[14].

Numerous genetic studies of aging in tissues of different species have revealed a number of common genes and signaling pathways that regulate organismal longevity, such as insulin-like growth factor and its components [2]–[5]. With the availability of microarrays, genome-wide transcriptional studies on aging have shown that different cell types with distinct functions share common signatures, and age similarly [6]–[9]. Rodwell et al. revealed that only a small fraction of transcriptional responses to age process are tissue-specific, suggesting that the molecular signatures of aging might overlap in different tissues [6]. Schumacher et al. found that similar expression changes were triggered constitutively in four organs of aged mice, also that both delayed and accelerated aging interventions could induce similar survival responses through shared, common longevity assurance mechanisms [7]. Recently, a meta-analysis of 27 age-related gene expression datasets from mice, rats and humans was performed to identify widely common signatures of aging [9]. Results from these genetic studies of aging suggest that a common set of molecular mechanisms may be responsible for the aging and lifespan of organism is subjected to certain genetic control.

Aging has also been was revealed to be a highly heterogeneous process at both the population and tissues level. Nonheritable variations in lifespan were found in laboratory populations of inbred lines of nematodes, fruit flies, and mice. Moreover, inbred worms and flies and outbred human populations, display multiphasic changes in mortality rates during aging [10]. Lu et al. investigated how human brains change between ages 26 and 106, and found that brain aging of people in their middle years exhibits great heterogeneity, with people appearing to approach old age at different speeds [11]. For organs or tissues within an organism, organs are known to age at very different speeds. One organ may show significant functional decline, while another retains appropriate performance. A study in *Caenorhabditis elegans* found a remarkable preservation of the nervous system, even in advanced old age, in contrast to a premature and progressive deterioration of muscle. This indicated the existence of extensive variability, both among same-age animals and between cells of the same type within individuals [12]. These studies clearly showed that different individuals or tissues of an organism age with different patterns, suggesting an underlying diversity of gene behaviors occurring within different types of cells with age.

Previous aging studies suggest that spatial gene changes determine lifespan in complex and paradoxical ways. We developed an analytical strategy to reconcile the commonality and heterogeneity of gene expression alteration in tissues during aging, to address the underlying molecular events in tissues associated with the various stages of life, and to investigate how this association evolves with age. The Significance Analysis of Spatio-Temporal Association of Genes Expression Alteration (SASTAGEA) is designed to detect tissue-associated changes in the expression of groups of age-related genes and reveal the temporal dynamic evolution of spatial gene association. The goal was gathering comprehensive and profound insights into the underlying molecular commonality and diversity through time and space during the aging process.

## Results

Throughout the lifespan, alterations in temporal gene expression in different tissues can be generalized as synchronous or asynchronous behaviors. For example, for a temporal process, if the same genes in different tissues present the same expression pattern, these genes exhibit synchronous behavior at the corresponding age phase and tissues; if they present the opposite or different expression direction, the genes exhibit asynchronous behavior. A global investigation of spatially synchronous and asynchronous gene expression occurring in different temporal processes may expose time-driven gene changes that are common or diverse among tissues. The significance of spatial association of genes expression actually represents intrinsic biological association between corresponding tissues. This will help us understand the underlying property of tissues coordination in age process.

### Dynamic evolution of spatial association of gene expression during aging

We first applied SASTAGEA to the natural aging process of three species: rat, mouse and *Drosophila melanogaster*. We conducted a natural aging experiment in rats as a genome-wide gene expression analysis from seven rat tissues, specifically hypothalamus (H), pituitary (P), adrenal (A), spleen lymphocyte (C), kidney (K), liver (L), and bone (B), measured at 4, 10, 18, and 24 months (see Material and Methods). Two additional public datasets on the natural aging process in *Drosophila melanogaster* and mouse were analyzed to provide more extensive species data. For *Drosophila*, the gene expression profiles from seven fly tissues, specifically muscle (MU), accessory gland (AG), brain (BR), testis (TE), malpighian tubule (MT), fat (FA) and gut (GU) were examined by measuring temporal changes in gene expression at in 15-, 20-, 30-, 45-,and 60-day-old flies, compared to 3-day-old flies [13]. For mice, gene expression profiles were measured for 16 dissected tissues, specifically cerebellum (CE), cerebrum (CR), striatum (ST), hippocampus (HI), spinal cord (SC), adrenal glands (AG), heart (HE), lung (LU), liver (LI), kidney (KI), muscle (MU), spleen (SP), thymus (TH), bone marrow (BM), eye (EY), and gonads (GO). Mice were 1-, 6-, 16-, or 24-months-old, with 10 mice per age cohort (five of each sex) [14].

**Figure 1:**
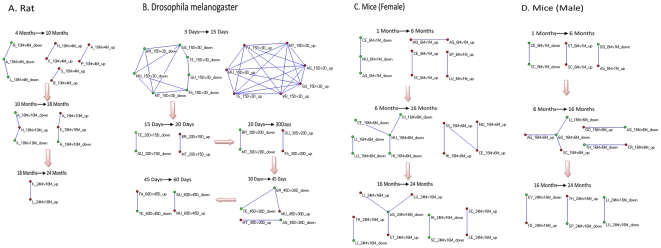
Temporal evolution of spatial association of gene expression during natural aging. The dynamic tissues association network of gene expression with age were illustrated respectively for (A) rat, p<0.001; (B) Drosophila melanogaster, p<0.005; (C) female mice, p<0.005; and (D) male mice, p<0.005. Red, gene set with increased expression at a certain age phase in a tissue; green, decreased expression. Node names incorporate tissue name,age phase, and expression direction. For example, the red node named A_10Mv4M_up (A) denotes an upregulated gene set from 4 to 10 months in adrenal gland (A). The connection between red (or green) nodes illustrates a significant number of genes synchronously increased (or decreased) in expression in the two connected tissues; red and green describe asynchronous behavior. For nodes not connected into networks, genes in the tissue lacked significant association with the others, reflecting a stochastic space relationship.

The dynamic evolution of the spatial association of gene expression with age in rats, mice, and *Drosophila* are illustrated in Figure 1. Nodes in the figure represent gene sets with expression that changed during a particular age phase in one tissue. Red nodes denote expression increase, and green nodes denote decrease. The name of the node denotes the represented tissue and age phase. For each age phase, if the number of overlapped genes between different nodes were significantly higher than random, (p-value lower than 0.005 or 0.001), nodes were connected into a network, showing a tissue association relationship. A connection between upregulated (or downregulated) nodes illustrates that a significant number of genes synchronously increased (or decreased) expression in two connected tissues. An upregulated or downregulated connection represents a significant number of genes asynchronously expressed in corresponding tissues (Figure 1).

For rats, we observed that a synchronous behavior dominated the spatial association relationship of gene expression from age 4 to 10 months. Furthermore, the asynchronous behaviors rose dramatically from age 10 to 18 months, while the synchronous behaviors greatly decreased. From 18 to 24 months of age, less tissue was connected, showing that the stochasticity of gene expression among tissues was aggravated by old age, and synchronous and asynchronous connections representing significant spatial association in gene expression nearly vanished (Figure 1A). For *Drosophila melanogaster*, a number of synchronous actions clearly emerged from 3 to 15 days, while a striking decrease in synchrony occurred between 15 and 30 days. With a further age increase, asynchronous behaviors distinctly rose between 30 and 60 days (Figure 1B).

For mice, a relatively large sample size across the lifespan was measured in both females and males, so we could separately analyze spatial changes in gene expression in different sexes, addressing gender-specific differences in aging that may exist at the level of gene activity. In female mice, the synchronous behavior was predominant from 6 to 16 months, as well as from 1 to 6 months. Asynchronous expression began to emerge in later life, at 16 to 24 months (Figure 1C). In male mice, a distinct difference in asynchronous behavior clearly occurred from 6 to 16 months (Figure 1D). Globally, across all mouse tissues, males showed earlier spatial asynchrony in gene activity than females. Clear gender differences in spatial gene expression association were evident, suggesting that the the mice body undergoes sexually dimorphic changes during the overall lifespan. Together, the evidence from *Drosophila*, rats, and mice demonstrated that age-related genes in different tissues or organs of an organism tend to show synchronous expression in young ages, but asynchronous expression in middle or late life, indicating that different aging tissues experience a regulatory evolution from commonality to imbalance or diversity with age. Furthermore, the number of genes with synchronous or asynchronous expression lost significance at middle or old age, suggesting enhanced spatial stochasticity of gene expression in old tissues.

### Anti-aging intervention restores spatial association of gene expression

Numerous studies have shown that either reduction in caloric intake, or an every-other-day feeding (EOD) of a nutritious diet, can delay the onset of age-related diseases and decelerate functional decline [15]–[18]. Resveratrol has also been shown to improve health, reduce signs of aging and extend the lifespan of different species including *Saccharomyes cerevisiae*, *C. elegans*, *D. melanogaster*, vertebrate fish, and mice [19]–[24]. Therefore, we applied our approach to a confirmed anti-aging intervention process performed by Pearson et al. [25]. This study examined the effects of resveratrol on mice fed a standard diet (SD) ad libitum, subjected to EOD, or fed a high calorie diet (HC) ad libitum. Initially, each dietary group was divided into no resveratrol (SD, EOD, HC), or low resveratrol (100 mg/kg of food, SDLR, EODLR). Later, additional groups of mice were given a higher dose of resveratrol with the standard diets (2400 mg/kg of food, SDHR and EODHR). Global gene expression profiles were measured to identify changes in transcriptional patterns for SD mice at 18 and 27 months of age for liver (L), muscle (M), fat (F) and heart (H). The corresponding gene expression was measured for HC, EOD, SDR, EODR, SDHR and EODHR mice at 27 months of age [25].

**Figure 2:**
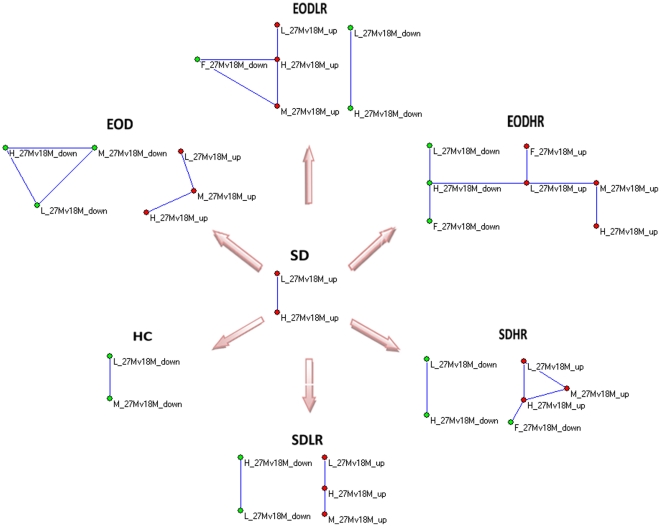
Reversion of spatial association of gene expression in aging tissue under the anti-aging intervention of caloric restriction or resveratrol treatment (p<0.01). The gene with expression alterations from 18 to 27 months in liver (L), muscle (M), adipose (A) and heart (H) from mice fed a standard diet with a low dose of resveratrol (SDLR) or a standard diet with high resveratrol (SDHR) showed significant restoration of spatial association compared to two reference groups of mice without anti-aging interventions fed a standard diet (SD) ad libitum or a high-calorie diet (HC) ad libitum, losing spatial association at 27 months. Similar restoration occurred in calorie-restricted mouse groups subjected to every-other-day feeding (EOD), every-other-day feeding with low resveratrol (EODLR), or every-other-day feeding with high resveratrol (EODHR).

We found that the spatial behavior of genes expression in mice fed only SD presented a similar pattern to those fed only HC, with the numbers of significantly connected tissues decreasing sharply from 18 to 27 months. This suggested an increase in spatial stochasticity of gene expression during old age (Figure 2). This was consistent with previous results observed in rat, mice, and *Drosophila* in old age. However, under anti-aging intervention by dietary restriction (EOD) and resveratrol (EODLR, EODHR, SDLR, SDHR), spatial connection and synchronous behavior among tissues was restored significantly from 18 to 27 months when compared to the groups without anti-aging intervention (SD, HC). This was coincidental with results observed in rats, mice, and *Drosophila* at a young age, suggesting a common molecular nature for the anti-aging action and young age (Figure 2). Both dietary restriction (EOD) and resveratrol may restore the spatial behavior of gene expression. We also saw that resveratrol treatment induced spatial transcriptional patterns resembling EOD feeding, suggesting that resveratrol mimics transcriptional effects of dietary restriction (DR) to some extent, providing some of the benefits of DR without a reduction in caloric intake. An anti-aging treatment might effectively improve functional decline and delay aging because it might modulate the underlying molecular mechanisms of the aging process. Thus, the results from confirmed anti-aging experiments support our findings.

### Synchronous and asynchronous expression with age of biological processes enriched for age-related genes

To investigate the biological context associated with age-related genes in synchronous or asynchronous expression among tissues, we used DAVID (Database for Annotation, Visualization, and Integrated Discovery) system [26], [27], a comprehensive and widely cited bioinformatics resources, to identify significantly enriched pathways and gene functional categories. We assembled genes with synchronous and asynchronous expression in each age phase, then extracted biological terms enriched by this genes set from several particular databases resources including Gene Ontology Biological Process (GO_BP), Swiss-Prot (SP), Protein Information Resource (PIR), BioCarta and KEGG pathway. The considered annotation terms here were first selected by the default threshold settings (at least containing two genes and having ease score less than 0.1) in DAVID system. Furthermore, those redundant terms in GO hierarchical structure and among databases were removed.

We first observed the common terms that appear at least twice in the three natural aging processes of female mouse, male mouse and rat (Table 1). Ease score (ES) and genes count of each term were presented accordingly. We found common terms were implicated mainly in several special biological categories including DNA/protein modification and repair (acetylation, phosphorylation, ubiquitinylation for example), cellular growth and differentiation (epithelial cell differentiation, pathways in cancer etc.), cellular response to stimulus (stress, hypoxia etc.), regulation of biological process (protein kinase cascade, gene expression etc.). Especially, the term ‘acetylation’ appeared in all of three natural aging groups, the ‘phosphorylation’ in female and male mouse groups. The two terms were enriched by relatively more genes compared with other terms. The specific genes list of each term was presented in table S1 that also contains some other annotation terms like GO cellular component (GO_CC). The result shows many genes in ‘acetylation’ were localized in mitochondrion (GO:0005739).

**Table 1:** Enriched functional terms for genes with synchronous and asynchronous expression commonly occurring in Rat and Mouse natural aging process.

Term(Database category)	Rat	Male Mouse	Female Mouse
	ES(count)	ES(count)	ES(count)
Acetylation(SP)	0.024(20)	0.0023(26)	<0.0001(41)
Phosphoprotein (Phosphorylation)(SP)	NA	<0.0001(62)	<0.0001(72)
Ubl conjugation (Ubiquitinylation)(SP)	NA	0.095(7)	0.094(8)
Response to hypoxia (GO_BP)	0.043(5)	0.0084(4)	NA
Cellular response to stress(GO_BP)	0.01(9)	0.049(7)	NA
Response to steroid hormone stimulus(GO_BP)	<0.0001(12)	0.058(3)	NA
Response to extracellular stimulus(GO_BP)	<0.0001(14)	NA	0.019(5)
Regulation of response to external stimulus (GO_BP)	0.025(5)	NA	0.045(4)
Striated muscle tissue development(GO_BP)	0.062(4)	0.05(4)	NA
Pathways in cancer(KEGG)	0.05(6)	NA	0.008(9)
Blood vessel development(GO_BP)	0.0052(7)	NA	0.00011(10)
Regulation of myeloid cell differentiation (GO_BP)	0.08(3)	NA	0.059(3)
Epithelial cell differentiation(GO_BP)	0.04(4)	NA	0.069(4)
Positive regulation of transcription (GO_BP)	0.077(8)	0.091(7)	0.030(9)
Positive regulation of cellular biosynthetic process (GO_BP)	0.088(9)	NA	0.026(10)
Regulation of protein kinase cascade (GO_BP)	0.027(6)	NA	0.032(5)
Positive regulation of gene expression (GO_BP)	0.035(9)	NA	0.035(9)
Negative regulation of protein kinase activity (GO_BP)	NA	0.043(3)	0.059(3)
Positive regulation of macromolecule metabolic process (GO_BP)	NA	0.055(9)	0.024(11)
Negative regulation of molecular function (GO_BP)	NA	0.055(4)	0.082(4)
Cell motion (GO_BP)	NA	0.092(6)	0.023(8)
Mitochondrial transport (GO_BP)	0.042(3)	NA	0.041(3)
Protein localization (GO_BP)	0.038(10)	NA	0.065(11)

Note: ES (count) here designates Ease Score and gene count of the term in DAVID system; ‘NA’ means the term is not significant annotation for this genes set; GO_BP designates the Gene Ontology Biological Process annotations; SP designates the set of keywords annotations drawn from the Swiss-Prot and Protein Information Resource databases; BioCarta and KEGG respectively denotes gene pathway annotations from the KEGG and BioCarta database.

We next explore enriched terms separately in natural aging processes of rat, female and male mouse, and observed genes expression change of those terms particularly from SP_PIR_Keywords, GO_BP, KEGG and BioCarta pathway databases. In female mice, the pathway terms from KEGG mainly include pathways in cancer, proteasome, Huntington's disease and Amyotrophic lateral sclerosis (ALS). The genes expression of these pathways synchronously decreased in adrenal glands (AG), muscle (MU) and cerebellum (CE) from 1 to 6 months, as well as in gonads (GO) and muscle (MU) from 6 to 16 months. In contrast, asynchronous expression of genes from above pathways occurred from 16 to 24 months in liver (LI) and thymus (TH) (Table S2). The same situation also dramatically appeared in those terms from other databases resource such as ‘ubl conjugation’, ‘acetylation’ and ‘mitochondrion’ etc. in SP_PIR_Keywords (Table S2). In male mice, some cancer-related pathways such as Wnt signaling pathway were enriched by genes with asynchronous expression from 6 to 16 months in adrenal glands (AG) and gonads (GO) (Table S3). Besides, pathways enriched for asynchronously expressed genes occurring in middle or late life also had a distinct characteristic. Many were involved in repairing damaged DNA including base-excision repair (BER), DNA repair, synthesis and replication pathway, as well as degrading unneeded or damaged proteins such as ubl conjugation. Genes in these pathways presented asynchronous expression from 6 to 16 months in AG and GO. The similar situation also occurred in terms related to DNA/protein modification like ‘acetylation’, ‘phosphorylation’ etc. (Table S3). These results indicated an unbalanced accumulation of DNA and protein damage in aged tissues, as well as unbalanced DNA repair and protein process functions.

In rats, the terms ECM-receptor interaction, focal adhesion, PPAR signaling pathway, and pathways in cancer from KEGG were significantly enriched by genes with synchronous expression from 4 to 10 months. A majority of genes showed down-regulated expression in adrenal glands (A), bone (B), kidney (K) and liver (L) (Table S4). The genes of terms from other databases mainly including acetylation (SP_PIR_Keywords), regulation of cell proliferation (GO:0042127), cellular response to insulin stimulus(GO:0032869) etc. also exhibited the same change feature (Table S4). While from 10 to 18 months, we observed many genes involved in above terms showed asynchronous expression among tissues. For example, ctnnb1 was overexpressed in the hypothalamus, but underexpressed in adrenal glands and kidney. Hif1a was underexpressed in hypothalamus, but overexpressed in kidney. RGD1309707 and Uba6 were overexpressed in the hypothalamus, but underexpressed in the kidney and adrenal gland, respectively. Mapt was overexpressed in the hypothalamus and underexpressed in the kidney, while Ndufa10 was underexpressed in the hypothalamus, and overexpressed in the kidney (Table S4).

After DR or/and resveratrol treatment, a large number of terms was significantly enriched for genes in synchronous expression. Table 2 lists common terms that were over-represented in at least two of three aging-delayed processes (EOD, SDLR and SDHR). We found DNA/protein repair and modification related terms ‘acetylation’, ‘phosphorylation’ and ‘ubiquitinylation’ that appeared commonly in natural aging process also dramatically occurred in aging-delayed processes. The ‘acetylation’ was detected in EOD, SDLR and SDHR groups, the ‘phosphorylation’ and ‘ubiquitinylation’ in EOD and SDLR. Besides, protein localization/transport terms and cellular component terms such as ‘mitochondrion’ and ‘cytoplasm’ also were over-represented in both natural aging and anti-aging processes. However, KEGG term ‘circadian rhythm’ and BioCarta ‘prion pathway’ associated with a group of fatal neurodegenerative diseases just appeared commonly in anti-aging processes, as well as some biological processes on energy, lipid, and amino acid metabolism from GO database. Table S5 lists specific genes of each term in table 2. Furthermore, we observed genes expression change of terms particularly from SP_PIR_Keywords, GO_BP, KEGG and BioCarta pathway databases respectively in EOD, SDLR and SDHR groups. The results shows observed terms were enriched just by a large number of genes with synchronous expression after anti-aging intervention (Table S6). For example, genes in the long-term potentiation showed synchronously increased expression in heart (H) and muscle (M) under both EOD and EODLR intervention, while genes involved in the neurotrophin signaling pathway, as well as in Alzheimer's, Parkinson's and Huntington's disease pathways showed synchronously decreased expression in heart (H) and liver (L) under SDLR treatment. Many genes in GO term ‘antigen processing and presentation of exogenous antigen’ (GO:0019884) were synchronously underexpressed in liver (L) and muscle (M) in the SDHR group (Table S6). Compared with corresponding genes expression in natural aging groups (SD) and accelerated aging group (HC), the anti-aging intervention enhanced the spatial synchrony of genes in affected terms (Table S6). These results suggested that confirmed anti-aging interventions recover the spatial balance of some molecular processes to some extent.

**Table 2:** Enriched functional terms for genes with synchronous expression commonly occurring in mouse anti-aging processes treated by dietary restriction (EOD) and resveratrol intervention (SDLR, SDHR).

Term(Database category)	EOD	SDLR	SDHR
	ES(count)	ES(count)	ES(count)
Acetylation(SP)	0.00012(26)	<0.0001(31)	0.00012(44)
Phosphoprotein (Phosphorylation)(SP)	<0.0001(55)	0.037(47)	NA
Ubl conjugation (Ubiquitinylation)(SP)	0.046(7)	0.085(7)	NA
Hydrolase(SP)	0.068(13)	0.095(14)	NA
Circadian rhythm(KEGG)	0.082(2)	NA	0.012(3)
Generation of precursor metabolites and energy(GO_BP)	NA	0.060(5)	0.018(8)
Cellular amino acid derivative metabolic process(GO_BP)	0.040(4)	NA	0.057(5)
Phospholipid metabolic process(GO_BP)	NA	0.065(4)	0.087(5)
Energy derivation by oxidation of organic compounds(GO_BP)	0.015(4)	NA	0.080(4)
Glycerolipid biosynthetic process(GO_BP)	NA	0.042(3)	0.021(4)
Lipid metabolism(SP)	NA	0.0082(5)	0.014(6)
Protein localization/transport(GO_BP)	0.048(9)	0.026(10)	0.088(13)
Transport(SP)	NA	0.0091(18)	0.098(23)
Transit peptide(SP)	NA	0.0013(10)	<0.0001(16)
Prion Pathway(BIOCARTA)	0.090(2)	0.098(2)	0.011(4)(KEGG)
Mitochondrion(SP)	NA	<0.0001(17)	<0.0001(22)
Cytoplasm(SP)	0.00056(29)	0.0070(29)	0.066(41)
Endosome(SP)	0.017(5)	0.028(5)	0.057(6)
Lysosome(SP)	NA	0.051(4)	0.00081(8)

Note: refer to table 1.

### Four-dimensional association reveals commonality and diversity in time and space

**Figure 3:**
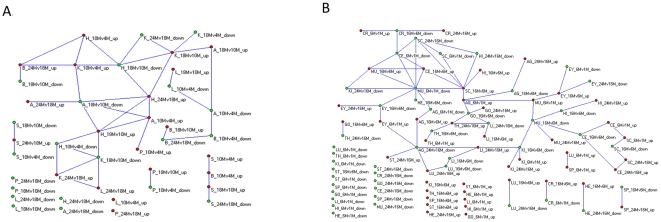
Four-dimensional genes association reveals commonality and diversity in time and space. The overall network of gene association through time and space were depicted for natural aging process of (A) male rats (p<0.0001), and (B) female mice (p<0.0005), showing age-related genes in particular age phases of tissues significantly occurring in another phase of the same or different tissues.

Since genes of a tissue may experience temporally dynamic expression with age, changed genes in one age phase might show the same or a different pattern at another age phase in the same tissue. For many tissues, imbalanced or asynchronous development with age might result in gene expression alteration during one age phase in one tissue occurring in another phase of another tissue with the same or a different pattern. Therefore, we tested whether a four-dimensional association through different time and space existed for age-related genes. In male rats and female mice (Figure 3), we observed that: i) most altered genes showed a temporally dynamic association in the same tissue. For example, genes with decreased expression from 1 to 6 months of age in mouse muscle (MU_6Mv1M_down) significantly increased expression from age 6 to 16 months (MU_16Mv6M_up) (Figure 3B); ii) a distinct gene association through time and space also widely occurred. For example in mice, a significant number of genes with downregulated expression from 1 to 6 months in muscle (MU_6Mv1M_down) also decreased expression from age 6 to 16 months in heart (HE_16Mv6M_down). However, expression increased in spinal cord (SC_16Mv6M_up) (Figure 3B); (iii) many genes expressed at a particular time and space exhibited only a tissue-specific temporal association. For example, genes with upregulated expression from 6 to 16 months of age in mouse lung (LU_16Mv6M_up) significantly showed decreased expression from age 16 to 24 months (LU_24Mv16M_down), but had no significant expression in any other tissue (Figure 3B); and iv) some genes showed only a spatio-temporal specific expression alteration. For example, the genes with decreased expression from 1 to 6 months of age in mouse lung (LU_6Mv1M_down) had no significant occurrence at any other time or space (Figure 3B). Similar results were observed in the aging process of male mice and *Drosophila melanogaster* (Figure S1). Connections between different time and space nodes revealed that some tissue at a particular age phase experienced a common or reversed molecular mechanism process than what occurred or will occur in another age phase of other tissues. This might be further demonstration of the asynchronous aging of tissues, indicating that tissues might age at different rates. Thus, the four-dimensional association maps of age-related genes gave us a comprehensive insight into intrinsic molecular commonalities and diversity through time and space.

## Discussion

In multicellular species, effective integrated functioning of the organism largely depends on sophisticated coordination among different tissues or organs. During aging process, the organismal function systematical and gradually decline. Therefore, an association exists between tissue coordination and aging, and investigating the evolution of tissue coordination with age is of interest. Theoretically, the tissues coordination could be associated with an underlying molecular network. Thus, exposing the spatial molecular representation during the various life stages should be a substantial approach for gaining insight into how tissues coordination evolves with age. In spite of previous research in this area, this is a poorly understood process. Our work on the dynamic spatial association behaviors of temporal gene expression alterations provides a novel angle for understanding tissue coordination. Our observations that spatially synchronous gene expression mainly occurred in young ages imply an accordant and harmonious regulation in diverse tissues during this phase. Subsequently, asynchrony dramatically increased in middle or old age, indicating that aging regulation of diverse tissues was inconsistent and incompatible, implying a decline of coordination function within the organism. In advanced old age, the significance of spatial association for both synchronous and asynchronous behaviors patterns almost disappeared, representing the aggravation of spatial stochasiticity of gene expression. Therefore, the evolution of tissue coordination with age could be a very complex process that still requires substantial works from various angles. Our results suggest the degree of coordination among tissues gradually becomes weaker with age, which might also be the reason for the decline in integrated function in older organism, and for the appearance of some age-related diseases. Our work provides a new perspective on aging.

Much aging research has tried to identify common aging signatures and a number of age-related genes that are common among tissues or species have been found [5]–[8]. However, many of these studies compared only two points, e.g., young and old, and the commonality of gene alterations in space may hide the intrinsic diversity of over time. For example, from our results with rats, we observed that Slc24a2 showed temporally dynamic expression values (5.53, 7.13, 7.09, 7.15) at four ages (4-, 10-, 18-, 24-months old) in adrenal glands, while the corresponding dynamic expression values in hypothalamus were 7.08, 7.24, 9.16 and 9.47. This showed that Slc24a2 in both adrenal gland and hypothalamus showed upregulated expression from 4 to 24 months (between young and old). However, Slc24a2 presented a diversity of temporally dynamic expressions in adrenal gland and hypothalamus, with increased expression at 10 months in adrenal gland, and at 18 months in the hypothalamus. Threfore, the common genes found in old and young concealed the temporally asynchronous appearance of the tissue aging process. Thus, a global investigation of gene association through time and space would be more helpful for gaining insight into the underlying molecular commonality and diversity among tissues during the aging process, and explain why both common and heterogeneous aging processes were found in previous studies.

Recently, many stochastic properties in the aging process have been investigated. For example, researchers showed that the expression of a variety of housekeeping and cell type-specific genes in individual murine cardiac myocytes became increasingly stochastic as the organism aged [28]. Similar stochastic effects were in aging murine muscle tissues [29]. Rea et al. found that the level of expression of a reporter expressed from a heat shock promoter in response to environmental stress on the first day of adulthood was remarkably stochastic. Moreover, this variation predicted the lifespan of the organism [30]–[31]. Inbred mice under essentially the same conditions demonstrated great variability in biological traits including body weight, and size, for which stochasticity accounted for 70–80% [32]. Our results that age-regulated genes with synchronous or asynchronous expression among tissues distinctly lost significance at old age revealed a new stochasticity property in spatial association of genome-wide gene expression. Genetic components have traditionally been thought to contribute greatly to the aging process, but current studies largely challenge this concept.

Although the underlying molecular mechanisms of aging remain a subject of debate, some anti-aging interventions including DR and resveratrol have been shown to delay aging to some extent in many species [15]–[24]. These age-delaying interventions may exert their effect through the underlying molecular mechanisms of aging. We observed that spatial gene expression was distinctly reversed from a stochastic behavior to a significantly synchronous or asynchronous association in old animals treated with DR and/or resveratrol. This result confirms our finding that the conversion of spatial gene expression from synchrony to asynchrony and stochasticity with age delineate a key molecular event in aging, reflecting the intrinsic mechanism of organismal aging.

A difference in aging between males and females is observed in various species. In most bird species, where the heterogametic sex is female (ZW sex chromosomes), males tend to live longer than females [33]–[34]. When the heterogametic sex (XY sex chromosomes) is male, as in humans and *Drosophila*, females tend to live longer than male. Berchtold et al. found clear gender differences in human brain aging, suggesting that the brain undergoes sexually dimorphic changes in gene expression not only in development but also later in life. Globally across all brain regions, males showed more gene changes than females [35]. We separately analyzed gene expression profiles for male and female mice, and found that the conversion of spatial gene expression association from synchrony to asynchrony occurred earlier in male mice than that in female mice, implying that male mice shows earlier aging traits.

Some studies of aging revealed time-dependent accumulation of DNA or protein damage in cells and organs, associated with gradual functional decline and aging [36]. Cancer and diseases of aging are two effects of DNA damage [37]–[38]. We found that functional pathway analysis of genes with synchronous and/or asynchronous expression at multiple ages demonstrated the same biological features during aging. Genes with asynchronous expression in middle or old age were enriched in pathways for DNA/protein modification, repairing or removing damaged DNA and degrading unneeded or damaged proteins. Pathways in cancer and some cancer-related pathways such as MAPK signaling and Wnt signaling were enriched in genes with asynchronous expression in tissues at middle or old age. These results suggested that asynchronous DNA/protein damage accumulation as well as asynchronous repair, modification and degradation of DNA/protein in tissues possibly promoted senescence or survival responses in tissue cells, leading to asynchronous and permissive alteration of the tissue microenvironment, structure and function. This increases risk for a variety of age-related diseases including atherosclerosis, neurodegeneration and cancer, eventually accelerates organismal aging and death. Interestingly, known age-delaying interventions reversed the asynchronous expression of genes in pathways related to DNA/protein damage, repair and modification, and pathways related to aging diseases including Alzheimer's, Parkinson's and Huntington's disease. This indicated that our findings represent an essential and conserved molecular event occurring in the aging process.

## Materials and Methods

### Microarray experiments of rat aging

This experiment in rats was carried out in strict accordance with the recommendations in the Guide for the Care and Use of Laboratory Animals of The Ministry of Science and Technology of the People's Republic of China. The protocol was approved by Office of Shanghai Administrative Committee for Lab. Animals, permit number SCXK(Hu) 2007-0005. Male Sprague-Dawley rats were housed in polycarbonate cages in a facility with automatically controlled temperature (22°C), humidity (50%) and light (12 h light/12 h dark cycle). Rats had free access to water and standard chow. We sampled seven tissues or organs, including hypothalamus, pituitary, adrenal gland, spleen lymphocytes, liver, kidney, and bone at four ages: 4-, 10-, 18-, and 24-months-old. For each age, 10 rats were obtained and 7 tissues from the same rat were used, with 5 µg of extracted RNA sample of the same tissue from 10 rats equally mixed. A sample of 15 µg RNA from this mixture was hybridized to Affymetrix RAE230A GeneChips. Thus, the rat RNA sample of each array came from 10 rats, allowing efficient coverage of genetic variations and minimizing the masking of intraindividual variability associated with the stochastic nature of aging [39]. Gene expression signals were calculated with MAS5.0 (Affymetrix) by global scaling to a target intensity of 500. All data is MIAME compliant and the raw data as well as processed data have been deposited in GEO database, accession number GSE23328.

### Other data collection

The other gene expression profiles from *Drosophila* and mice were respectively collected from the following sources: (a) the normalized data of natural aging in *Drosophila melanogaster* was downloaded from GEO at http://www.ncbi.nlm.nih.gov/geo/, accession number GSE6314 [13]; (b) The normalized and processed data of normal aging in male and female mice was directly obtained from the AGEMAP website at http://cmgm.stanford.edu/~kimlab/aging_mouse [14]. Here, we removed bone marrow samples in female mice because they had less than three biological repeats for the 24-month age group; (c) Normalized data of the anti-aging process under intervention of resveratrol and dietary restriction in mice was also obtained from GEO, accession number GSE11845 [25].

### Gene probes filtering

To eliminate gene probes that were potentially unreliable or had noise effects, and to facilitate comparisons, analysis of all datasets was restricted to genes complying with the following two criteria: i) all genes probes with missing values greater than 80% of the total number of samples were removed; and ii) replicated probes representing the same gene were excluded, leaving only the probe that has the maximum number of samples with presented expression.

### Detecting temporal change in gene expression

Gene expression alteration for every temporal phase of each tissue was calculated. Because we used datasets from different microarray platforms and designs, we adopted different statistical approaches to detect differentially expressed genes. For the rat aging dataset measured by Affymetrix gene chip, we combined statistical change algorithms from MAS5.0 and fold-change to find altered genes. Briefly, comparisons used three filters: changed p-values <0.0025 for expression increase (1- change p-values <0.0025 for expression decrease), absolute fold-change of at least 2, and two samples for comparison all detected as present (P) by the MAS 5.0 detection call algorithm. The mouse normal aging dataset had data subjected to Z score normalization transformation, and contained at least three replicated arrays for each tissue, sex and age class. Therefore, an alternative method for calculating significant changes in gene expression was the one-sided two-sample Z test (p<0.01), which maximizes the power of replicates and accounts for variation between replicates on a gene-by-gene basis. Z-tests were also used for the mouse dataset under anti-aging intervention, which used the same Z-score transformation of raw data (p<0.001). For *Drosophila*, two sets of DNA microarray data for the same aging process were measured. We selected genes with a fold-change of at least two for both datasets.

### Spatial association significance of gene expression with the temporal progress of aging

When gene expression increased or decreased in a tissue during a temporal phase, we needed to know if it also increased or decreased in another tissue during the same phase. To determine significant genes with a certain expression behavior between tissues (T_i_ and T_j,_) at a particular age phase P_k_, we denoted N as the number of all genes on the chip, M as the number of genes upregulated or downregulated in tissue T_i_, K as the number of upregulated or downregulated genes in tissue T_j_, and x as the number of overlapped genes with a certain expression behavior between tissues T_i_ and T_j_. The probability that tissue T_i_ has x upregulated or downregulated genes by chance from the list of upregulated or downregulated genes in tissue T_j_ was modeled by a hypergeometric distribution with parameters (N, M, K). The p-value of having x genes or more in tissue T_i_ was calculated by summing the probabilities of a random list of K genes having 0,1, 2, … , x-1 genes in tissue T_i_:
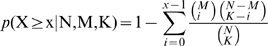
Pairwise tissue comparisons were calculated and tissue pairs with a p-value less than 0.005 or 0.001 were connected into a network as a significant association. We can create a network for each age phase to show the dynamic evolution of the tissue association of gene expression with aging. A global four-dimensional association network through time and space was built by comparing all gene sets under different temporal and spatial situations, comparing changed genes from tissue T_i_ in phase P_n_ with the genes from tissue T_j_ in phase Pm. This comprehensively reflected synchronism, asynchronous, and specific behavior of gene expression among tissues over the lifespan.

## Supporting Information

Table S1 **Enriched functional terms for genes with synchronous and asynchronous expression commonly occurring in Rat and Mouse natural aging process.**
(XLS)

Table S2 **Enriched functional terms of age-related genes with synchronous and/or asynchronous expression during the natural aging process of female mouse.**
(XLS)

Table S3 **Enriched functional terms of age-related genes with synchronous and/or asynchronous expression during the natural aging process of male mouse.**
(XLS)

Table S4 **Enriched functional terms of age-related genes with synchronous and/or asynchronous expression during the natural aging process of rats.**
(XLS)

Table S5 **Enriched functional terms for genes with synchronous expression commonly occurring in anti-aging processes of mouse respectively treated by dietary restriction (EOD) and resveratrol intervention (SDLR,SDHR).**
(XLS)

Table S6 **Enriched functional terms of age-related genes in synchronous expression under anti-aging caloric restriction and/or resveratrol.**
(XLS)

Figure S1 **Overall network of gene associations through time and space in the aging process of (A) male mice (p<0.0005) and (B)** ***Drosophila melanogaster*** **(p<0.0005), showing age-related genes occurring in a particular age phase of a tissue significantly occurring in another phase of the same or different tissue.**
(PDF)
