# Supplementary material for: The Spatial Association of Gene Expression Evolves from Synchrony to Asynchrony and Stochasticity with Age
Source: PLoS One. 2011 Sep 2;6(9):e24076. doi: 10.1371/journal.pone.0024076 (PMC3166296; doi:10.1371/journal.pone.0024076)
Supplement: Figure S1 — Overall network of gene associations through time and space in the aging process of (A) male mice (p<0.0005) and (B) Drosophila melanogaster (p<0.0005), showing age-related genes occurring in a particular age phase of a tissue significantly occurring in another phase of the same or different tissue. (PDF) [file pone.0024076.s007.pdf]

A

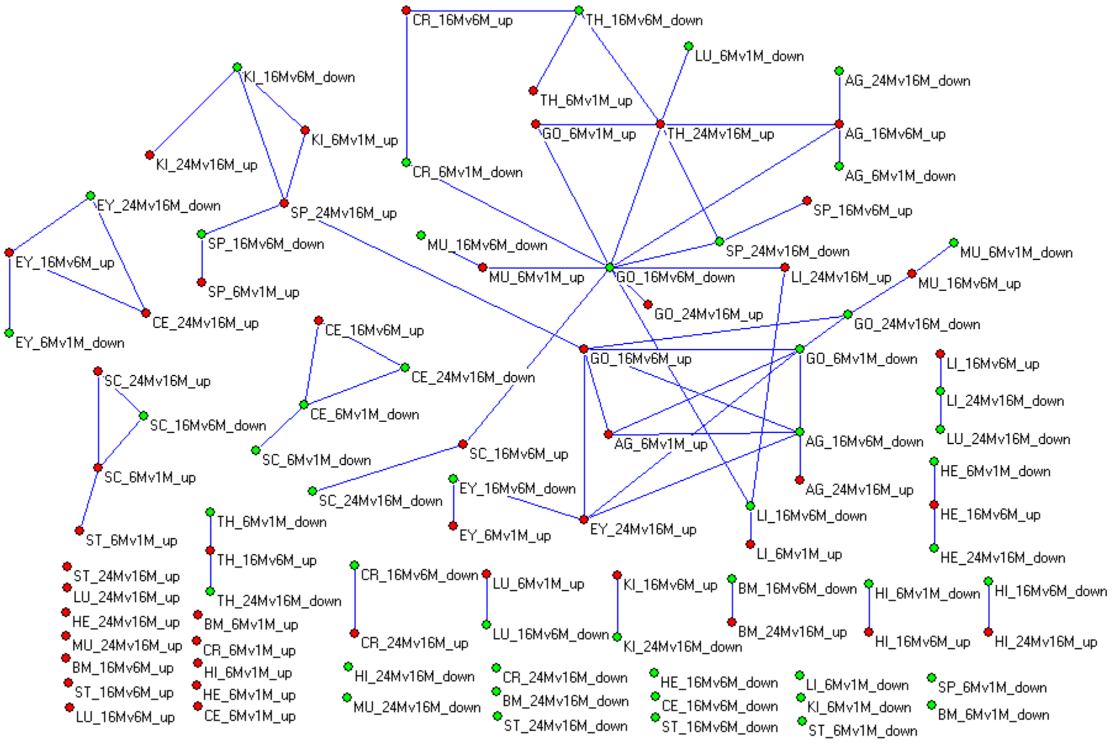

B

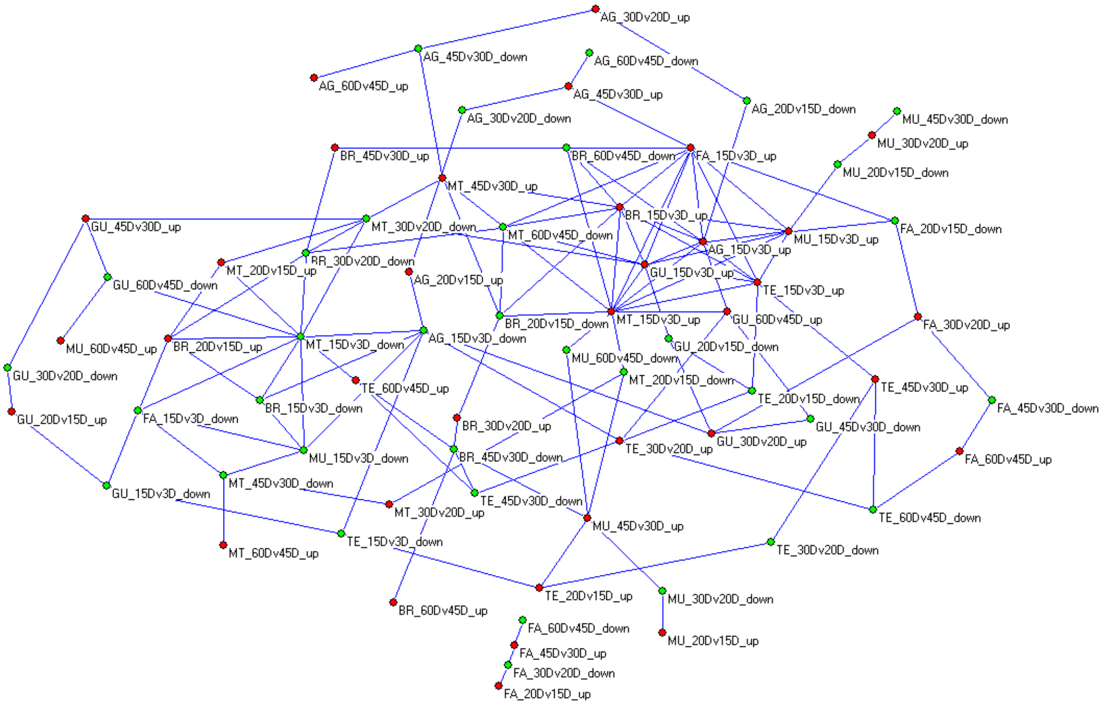

Figure S1 illustrates overall network of genes association through different time and space in age process of (A) male Mice($p<0.0005$) and (B) Drosophila melanogaster($p<0.0005$), showing whether the age-related genes occurring in particular age phase of some tissue at number significantly also occur in another phase of the same or different tissue.
